# Complete Genome Sequence of *Pseudomonas* sp. Strain SK, Isolated from Organic Wheat Rhizosphere

**DOI:** 10.1128/MRA.00510-20

**Published:** 2020-06-25

**Authors:** Shivani Khatri, Pavelas Sazinas, Yashbir S. Shivay, Shilpi Sharma, Lars Jelsbak

**Affiliations:** a Department of Biochemical Engineering and Biotechnology, Indian Institute of Technology Delhi, Hauz Khas, New Delhi, India; b Department of Biotechnology and Biomedicine, Technical University of Denmark, Kongens Lyngby, Denmark; c Division of Agronomy, ICAR–Indian Agricultural Research Institute, New Delhi, India; Loyola University Chicago

## Abstract

Here, we report the annotated whole-genome sequence of *Pseudomonas* sp. strain SK, isolated in India from organic wheat rhizosphere. This strain has proved to be a species with potential biocontrol activity against soilborne plant pathogens based on antiSMASH analysis.

## ANNOUNCEMENT

The fluorescent *Pseudomonas* strains present in rhizospheric soil play a critical role by combating fungal and bacterial pathogenic stress through the production of several metabolites and antibiotics (1). In this study, we have isolated and sequenced a fluorescent *Pseudomonas* sp. from the rhizosphere of wheat grown in organically amended soil.

*Pseudomonas* sp. strain SK was isolated by serial dilution of rhizospheric soil samples, followed by plating onto specific *Pseudomonas* isolation agar base (HiMedia, India) with overnight incubation at 30°C. To confirm that this strain belongs to the genus *Pseudomonas*, 16S rRNA and *rpoD* gene sequencing were carried out using the following primer sets: universal primers for 16S rRNA genes 8F (5′-AGAGTTTGATCCTGGCTCAG-3′) and 1492R (5′-GGTTACCTTGTTACGACTT-3′) and the *rpoD* gene primers PsEG30F (5′-ATYGAAATCGCCAARCG-3′) and PsEG790R (5′-CGGTTGATKTCCTTGA-3′). Genomic DNA was isolated with the PureLink genomic DNA kit (Thermo Fisher Scientific). For Illumina sequencing, DNA libraries were generated using a modified protocol (half volume of reagents) of the KAPA HyperPlus library preparation kit (Roche Molecular Systems), and these were then sequenced on the Illumina MiSeq instrument (2 × 150 bp). A total of 3,599,278 paired-end reads were produced and subsequently trimmed based on quality (Q20) using Trim Galore v0.6.5 with the setting “-q 20” (https://github.com/FelixKrueger/TrimGalore). For Nanopore sequencing, DNA libraries were prepared with the ligation sequencing kit (Oxford Nanopore) and sequenced on the MinION instrument with a FLO-MIN106 flow cell. Using MinKNOW v19.12.2 (Oxford Nanopore), 216,185 reads with a value above Q7 were basecalled. The reads had an average length of 3,098 bp and an *N*_50_ value of 7,555 bp. Unicycler v0.4.6 was used to carry out a hybrid genome assembly from both the Illumina and Nanopore reads using default parameters (2). A complete circular genome was assembled, with a length of 5,670,001 bp, a G+C content of 63.28%, and average genome coverage of 90.6× (for Illumina reads) and 113.6× (for Nanopore reads). The genome was annotated with the NCBI Prokaryotic Genome Annotation Pipeline v4.11 and was predicted to have 5,023 protein-coding genes, 78 tRNAs, 4 noncoding RNAs, 20 rRNAs, and 53 pseudogenes (3). To attempt a taxonomic assignment, we first performed a BLASTN search for a concatenated sequence of four housekeeping genes, *rpoD*, *rpoB*, *gyrA*, and 16S rRNA, against the nonredundant nucleotide database (4). The genome sequences of the 10 highest BLASTN hits were considered further for average nucleotide identity analysis using the JSpeciesWS online tool (5). There were no hits above the species assignment threshold (95%), suggesting that *Pseudomonas* sp. strain SK belongs to a previously uncharacterized *Pseudomonas* species.

The biocontrol activity of *Pseudomonas* sp. strain SK was confirmed by genome mining for potential secondary metabolites using antiSMASH v5.1.2 (6). The antiSMASH results showed seven potential secondary metabolite clusters, of which four belong to nonribosomal peptide synthetase, one to bacteriocins, one to *N*-acetylglutaminylglutamine amide, and one to homoserine lactone.

### Data availability.

The complete genome sequence of *Pseudomonas* sp. strain SK has been submitted to GenBank with the accession number CP051857 under the BioProject number PRJNA624785. The raw sequence reads generated with Oxford Nanopore and Illumina MiSeq have been deposited with the SRA numbers SRR11528729 and SRR11528730, respectively.
